# Whole genome sequence data of *Erwinia amilovora* strain E22, from Kazakhstan

**DOI:** 10.1016/j.dib.2024.111090

**Published:** 2024-10-28

**Authors:** Amankeldi Sadanov, Elvira Ismailova, Madina Alexyuk, Olga Shemshura, Gul Baimakhanova, Baiken Baimakhanova, Zere Turlybaeva, Assel Molzhigitova, Akmeiir Yelubayeva, Diana Tleubekova, Andrey Bogoyavlenskiy

**Affiliations:** Research and Production Center for Microbiology and Virology, Bogenbay batyr. Str., 105, Almaty 050010, Kazakhstan

**Keywords:** *Erwinia amilovora*, Whole genome, Kazakhstan, Genome annotation, Phylogeny

## Abstract

*Erwinia amilovora* is the causative agent of bacterial blight of rosaceae plants. The disease affects ornamental species of this family and fruit trees of great economic importance, such as apple and pear. In the presented research, sequencing of the *Erwinia amilovora* strain E22 isolated in Kazakhstan, was performed on the Illumina MiSeq platform, followed by bioinformatics processing and gene annotation using SPAdes, RAST, antiSMASH and CARD programs and databases. The size of the assembled genome is 3,799,623 bp. Annotation of the *Erwinia amilovora* genome assembly identified 3462 genes, including 3251 protein-coding genes and 117 RNA genes. This genome will be helpful to further understand the evolution of *Erwinia amilovora* and can be useful for obtaining control agents.

Specifications Table

**Table:** 

Subject	*Microbiology: Bacteriology*
Specific subject area	*Microbial genomics*
Type of data	Raw and processed sequencing data, genome annotation and phylogenetic analysis. Table, Graph, Figure.
Data collection	*Collection: The sample was obtained from the affected stem of the apple tree. The sample was cultured on King medium B. Single colonies observed after 48 h of incubation on King's medium B at 27 °C were isolated and subsequently purified. Total DNA was extracted from an overnight culture grown at 25 °C in LB medium using the the GeneJET Genomic DNA Purification Kit. The integrity of gDNA was checked by agarose gel electrophoresis and quantified using a Qubit 3.0 fluorometer. The sequencing libraries were then prepared using Nextera XT library preparation kit. The Miseq Illumina sequencing platform was used to generate paired-end reads. Software: The genome sequence* *was assembled using SPAdes assembler in Geneious Prime. Genome* *sequences were annotated using the GenBank PGAP annotator robot and* *deposited at NCBI. antiSMASH program and Comprehensive Antibiotic Resistance Database were used for determination of gene clusters synthesizing secondary metabolites and antibiotic resistance genes. The phylogenetic tree was constructed using FastTree Plugin in Geneious Prime.*
Data source location	*Research and Production Center for Microbiology and Virology, Almaty, Kazakhstan.* • City/Region: Almaty • Country: Kazakhstan • Latitude and longitude for collected samples: 43°15′14.2"N 76°57′11.1"E
Data accessibility	Repository name: GenBank: Data identification numbers: BioProject Accession Number: PRJNA224116, NCBI SRA Accession Number: SRX25261673, NCBI GenBank Accession Number: NZ_CP158121.1 The direct URL to the data: https://www.ncbi.nlm.nih.gov/bioproject/1120602 https://www.ncbi.nlm.nih.gov/sra/?term=SRX25261673 https://www.ncbi.nlm.nih.gov/nuccore/NZ_CP158121

## 1 Value of the Data

• The data of whole genome sequencing of *Erwinia amilovora strain E22*, isolated on the territory of the Republic of Kazakhstan are presented for the first time. The obtained data are publicly available in the NCBI databases, which expands the volume of genetic information on *Erwinia amilovora* isolates and contributes to a better understanding of the population structure of strains circulating in Central Asia.
• The presence in the microorganism genome structure of clusters responsible for the synthesis of secondary metabolites and genes encoding resistance factors to antibiotics of different groups has been shown, which gives advantages to the studied strain of *Erwinia amilovora* in spreading.
• The presented data can contribute to a better understanding of the evolutionary variability of *Erwinia amilovora* strains, the development of genetic markers for the diagnosis of pathogens and its adaptive capabilities due to the production of secondary metabolites to overcome the host's defense systems and survive in adverse conditions.
• The presented data may be useful to the scientific community in research aimed at developing methods for the prevention and treatment of bacterial blight of cultivated and wild plants of the Rosaceae family.

## 2 Background

Fire blight of fruit crops is a dangerous infectious disease of cultivated and wild plants of the Rosaceae family, caused by a *Erwinia amilovora* bacterium [1]. The first cases of bacterial blight were recorded in New York State at the end of the 18th century [2]. The disease spread to Canada, Mexico, Chile, and Guatemala. In the mid-1950s, the disease was first noted on pears in Kent, England [3]. In Poland, the disease was detected on pear trees in 1966 [4]. After 2005, fire blight lesions were observed in most countries of the European Union - from Cyprus in the south, to Sweden in the north, as well as outside the European Union (Armenia, Egypt, Israel, Jordan, Lebanon, Norway, Switzerland, Turkey, Ukraine, Belarus, Russia, Kazakhstan, China) [[4], [5], [6], [7], [8]].

The article describes the genetic characteristics of the complete genome of the *Erwinia amilovora*strain E22 isolated in Kazakhstan.

## 3 Data Description

**Fig. 1:**
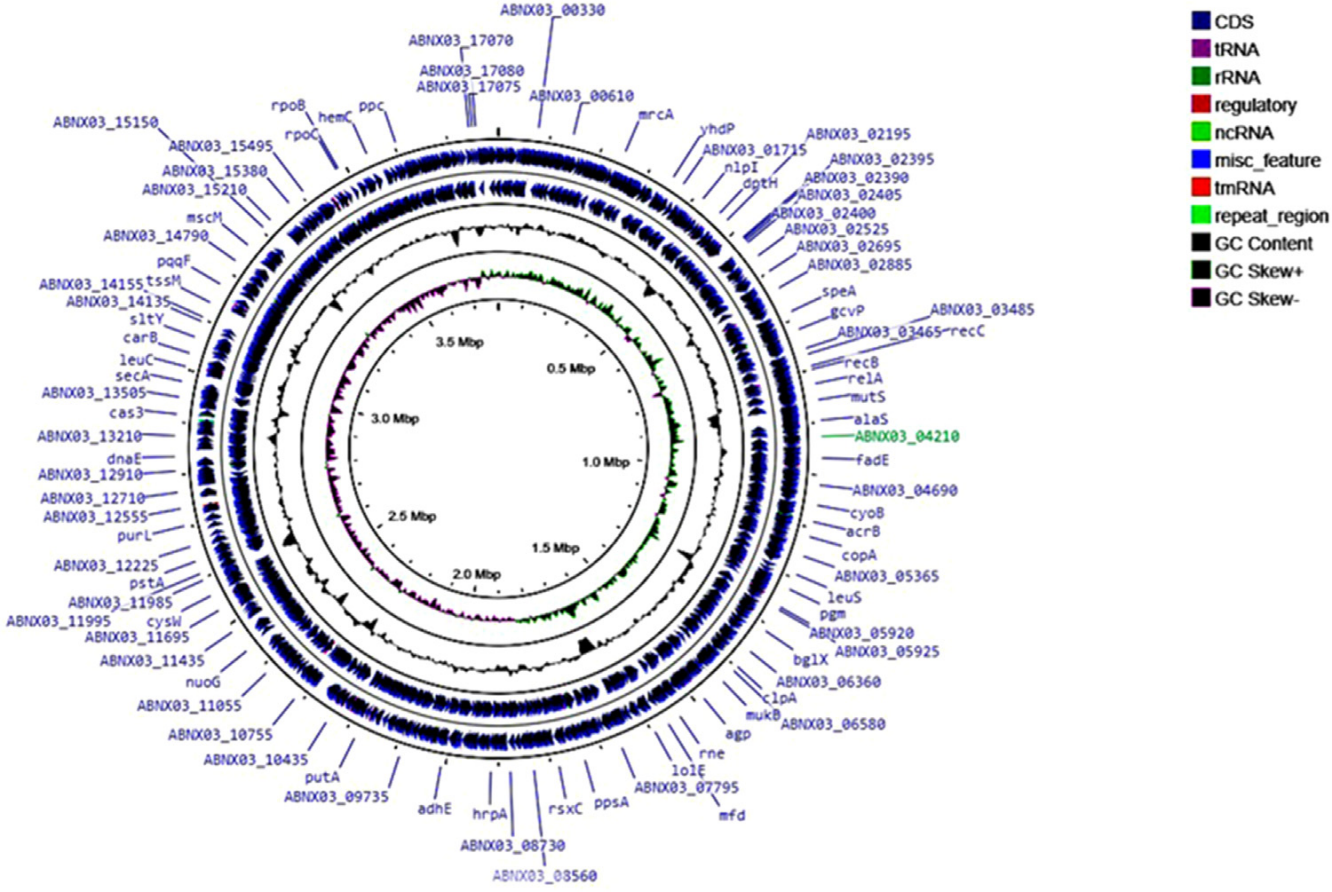
The genome map of *Erwinia amilovora strain E22*. The blue arrows represent CDSs; green peaks represent GC-skew+; purple represents GC-skew-; and black peaks represent G+C content.

**Fig. 2:**
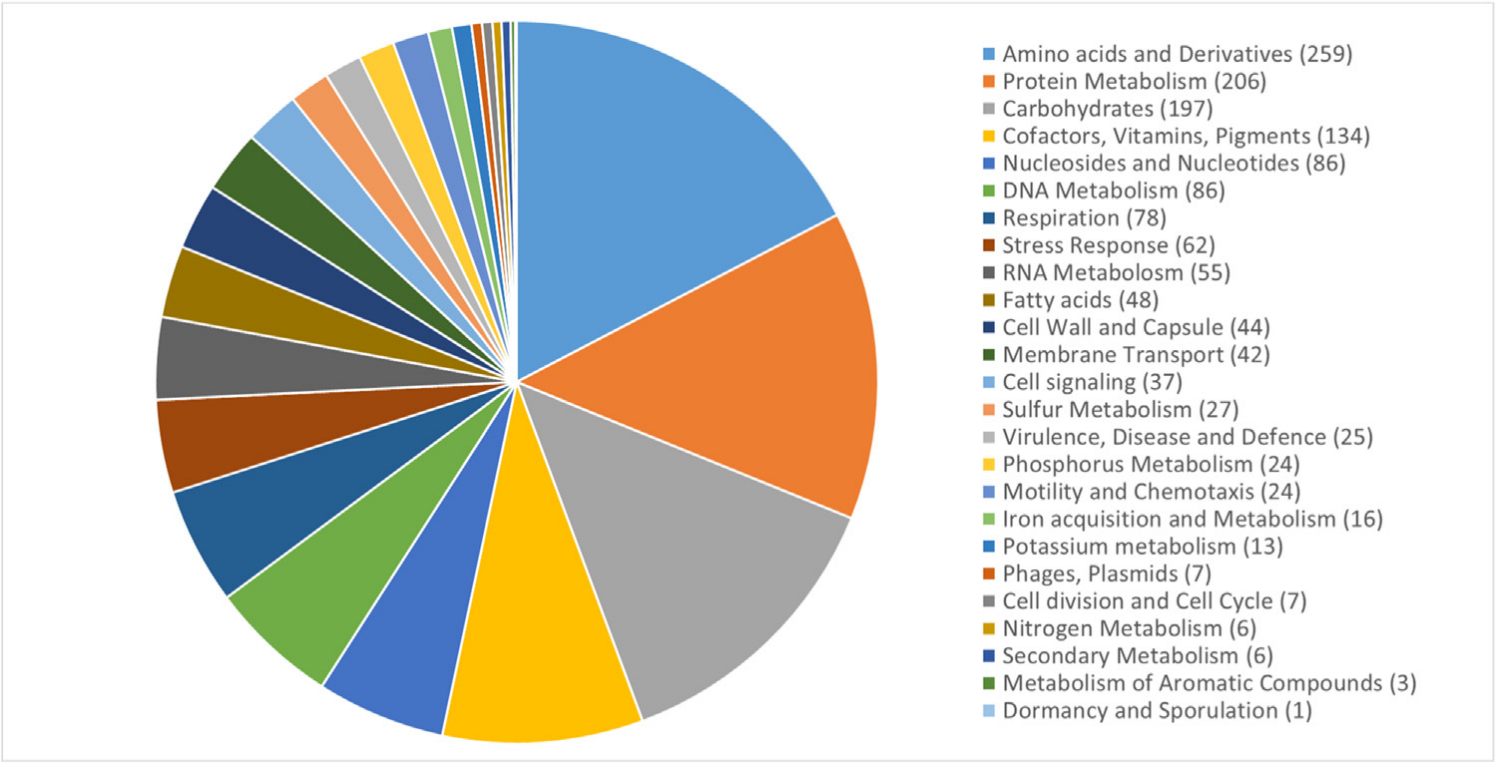
Subsystem statistics information on genome *Erwinia amilovora strain E22* obtained using RAST annotation. The subsystems categories and corresponding counts are presented in the legend.

This work presents a draft genome sequence of *Erwinia amilovora* strain E22. This strain was isolated from the affected organs of the Golden Delicious apple tree in Almaty region, Kazakhstan. The presented genome is a total of 3,799,623 bp long and contains 3462 genes, including 117 RNA genes. Based on genome annotation in the RAST 25 functional groups of genes were identified (Fig. 2). The largest number of which belonged to the following groups: Amino Acids and Derivatives, Protein Metabolism, Carbohydrates and Cofactors, Vitamins, Pigments (Table 1, Figs. 1, 2).

**Table 1:** Genome characteristics of *Erwinia amilovora* strain E22.

Genome size (bp)	3,799,623 (3,8 Mb)
GC% content	53.5 %
Number of contigs	1
N50 contig	1
Genes (total)	3462
CDSs (total)	3251
Genes (RNA)	117
tRNAs	76
CRISPR Arrays	2

**Fig. 3:**
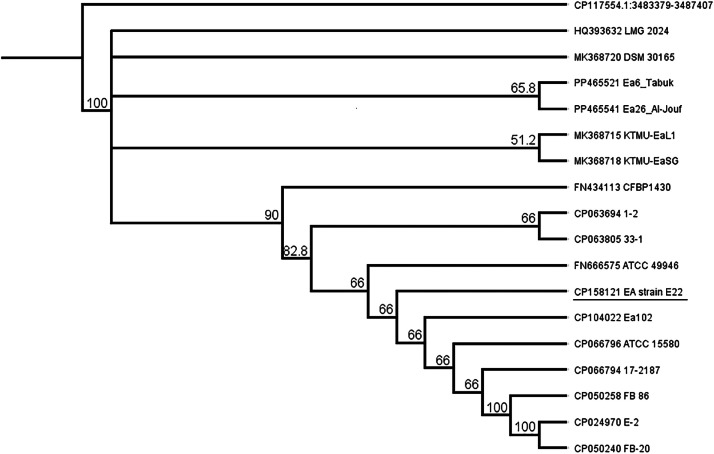
Phylogeny strains of *Erwinia amilovora* from different geographic regions on the model of RNA dependent DNA polymerase gene.

To perform phylogenetic analysis, the sequence of RNA dependent DNA polymerase gene was used (Fig. 3). It is shown that phylogeny E. amilovora E22 is closely related to the *Erwinia amilovora* type strain FB-20 (CP050240) from South Korea and strain Ea102 (CP104022) from China. Phylogenetic analysis of strains of the microorganism from different geographical regions showed that the isolated strain forms one group with strains from Russia and Asia.

Additionally, in the genome *Erwinia amilovora strain E22*, the presence of gene clusters synthesizing secondary metabolites and antibiotic resistance genes was determined by antiSMASH 7.0 program and Comprehensive Antibiotic Resistance Database (CARD; card.mcmaster.ca). Five gene clusters and 10 antibiotic resistance genes were determined (Tables 2, 3).

**Table 2:** Predicted gene clusters synthesizing secondary metabolites of *Erwinia amilovorastrain E22*.

Region	Type	From	To	Most similar known cluster	Similarity
Region 1	NRPS,T1PKS	500,492	554,217	anthelvencin A/anthelvencin B/anthelvencin C	13 %
Region 2	RiPP-like	1,410,186	1,421,943		
Region 3	NRPS	2,565,403	2,626,480	rhizomide A/rhizomide B/rhizomide C	100 %
Region 4	RRE-containing	3,165,156	3,185,377	basiliskamide A/basiliskamide B	9 %
Region 5	NI-siderophore	3,322,895	3,353,249	desferrioxamine E	75 %

**Table 3:** Predicted antimicrobial resistance genes of *Erwinia amilovora strain E22*.

RGI Criteria	ARO Term	Detection Criteria	AMR Gene Family	Drug Class	Resistance Mechanism	% identity of Matching Region
Strict	*CRP*	protein homolog model	resistance-nodulation-cell division (RND) antibiotic efflux pump	macrolide antibiotic, fluoroquinolone antibiotic, penam	antibiotic efflux	98.57
Strict	*Klebsiella pneumoniae KpnF*	protein homolog model	small multidrug resistance (SMR) antibiotic efflux pump	macrolide antibiotic, aminoglycoside antibiotic, cephalosporin, tetracycline antibiotic, peptide antibiotic, rifamycin antibiotic, disinfecting agents and antiseptics	antibiotic efflux	77.06
Strict	r*smA*	protein homolog model	resistance-nodulation-cell division (RND) antibiotic efflux pump	fluoroquinolone antibiotic, diaminopyrimidine antibiotic, phenicol antibiotic	antibiotic efflux	85.25
Strict	*ArnT*	protein homolog model	pmr phosphoethanolamine transferase	peptide antibiotic	antibiotic target alteration	58.47
Strict	*emrR*	protein homolog model	major facilitator superfamily (MFS) antibiotic efflux pump	fluoroquinolone antibiotic	antibiotic efflux	84.21
Strict	*Klebsiella pneumoniae KpnH*	protein homolog model	major facilitator superfamily (MFS) antibiotic efflux pump	macrolide antibiotic, fluoroquinolone antibiotic, aminoglycoside antibiotic, carbapenem, cephalosporin, penam, peptide antibiotic, penem	antibiotic efflux	84.09
Strict	*Morganella morganii gyrB* conferring resistance to fluoroquinolones	protein variant model	fluoroquinolone resistant gyrB	fluoroquinolone antibiotic	antibiotic target alteration	80.47
Strict	*Escherichia coli EF-Tu* mutants conferring resistance to Pulvomycin	protein variant model	elfamycin resistant EF-Tu	elfamycin antibiotic	antibiotic target alteration	94.66
Strict	*Haemophilus influenzae PBP3* conferring resistance to beta-lactam antibiotics	protein variant model	Penicillin-binding protein mutations conferring resistance to beta-lactam antibiotics	cephalosporin, cephamycin, penam	antibiotic target alteration	51.58
Strict	*Escherichia coli EF-Tu* mutants conferring resistance to Pulvomycin	protein variant model	elfamycin resistant EF-Tu	elfamycin antibiotic	antibiotic target alteration	95.17

Despite significant efforts to control bacterial blight worldwide, it continues to cause significant yield losses and tree mortality. The overuse of chemical plant protection products against bacterial blight observed in the past has led to a number of negative consequences: the development of resistant strains of pathogens and the accumulation of toxic residues in the environment. Therefore, determining the structure of the complete genome of newly isolated *Erwinia amilovora* strains is important for studying the evolution and distribution of this group of microorganisms. In our study, we presented for the first time a draft genome of *Erwinia amilovora* strain E22, isolated from the affected organs of Golden Delicious apple trees in Kazakhstan. The peculiarity of the studied strain is that 10 antibiotic resistance genes with different mechanisms of action and 5 clusters of synthesis of secondary metabolites contributing to the suppression of soil actinomycetes were detected in its genome.

Thus, the draft genome of *Erwinia amilovora* strain E22 may serve as an additional tool for researchers studying the genetic diversity of *Erwinia amilovora*, including strains circulating in Kazakhstan for the diagnosis of bacterial blight and, ultimately, the development of biologics for its treatment.

## 4 Experimental Design, Materials and Methods

### 4.1 Sample Collection and Isolation of *Erwinia Amilovora*

During monitoring surveys of orchards in the south and southeast of Kazakhstan in 2022, the causative agent of fire blight E. amylovora E22 was isolated from the affected organs of the Golden Delicious apple tree (Almaty region, Kazakhstan). From the affected stems suitable for analysis and prewashed for 20 min with tap water, a piece was cut out at the border of diseased and healthy tissue. The isolated piece of tissue was placed in sterile water, thoroughly grinded in a mortar and the macerate was applied to the surface of sucrose-peptone agar (SPA) nutrient medium in Petri dishes. The dishes were then placed in a thermostat at 28–30 °C. After 48 h of incubation, bacterial growth was checked and colonies similar to *Erwinia amilovora* were transferred to King's slope agar. If the bacterial isolate stained King's medium fluorescent green, then it belonged to the Pseudomonas genus. Bacteria of the genus Erwinia do not produce such a fluorescent pigment on this medium [9].

In general, culture on three media is recommended to maximize the likelihood of isolating E. amylovora. Depending on the number and composition of microorganisms in the sample, each medium may be more or less effective (SPA, King and Levan medium) [9].

E. amylovora forms round, milky-colored, shiny colonies on SPA nutrient medium. Colonies on King B medium are creamy-white, rounded, and do not fluoresce in UV light at 366 nm. Colonies of E. amylovora on Levan medium are white, rounded, dome-shaped, smooth and mucoid.

The pathogenic properties of isolated bacteria were tested using Clement's infectious-infiltration method using a hypersensitivity reaction to indicator plants of indoor geranium (Pelargonium zonale L.) (L'Hér.) or on tobacco leaves (Nicotiana tabacum L) and on young unripe pear fruits using White's method [10].

### 4.2 DNA Isolation, Genome Sequencing, Assembly, and Annotation

Genomic DNA was isolated using the GeneJET Genomic DNA Purification Kit according to the manufacturer's instructions (ThermoScientific, Waltham, MA, USA). A whole genome sequencing library was prepared using the Nextera XT DNA library preparation kit following the manufacturer's instructions (Illumina, Cambridge, UK).

The libraries were sequenced using the Miseq platform (Illumina, Cambridge, UK) to generate 2 × 300 paired end reads. The raw reads adapters were trimmed by Trimmomatic version 0.38.0 [11]. Low-quality sequences (<Q30) were deleted. After trimming, reads were contained from 50 to 250 bp. Genome assembly was performed using SPAdes version 3.12.0 [12]. After assembly, the quality of the genome was checked by the software Geneious Prime 2023 by mapping to the reference genome [13]. The annotation genome was determined by the NCBI Prokaryotic Genome Annotation Pipeline (PGAP), GeneMarkS-2+, RAST [14]. The the presence of gene clusters synthesizing secondary metabolites and antibiotic resistance genes was determined by antiSMASH 7.0 program and Comprehensive Antibiotic Resistance Database (CARD; card.mcmaster.ca) [15,16]. Phylogenetic tree was generated using FastTree Plugin default settings in Geneious Prime based on the model of the RNA dependent DNA polymerase gene of microorganism strains isolated in different geographical regions.

The raw genome sequencing data of Illumina MiSeq were submitted to NCBI SRA database in FASTQ format: SRX25261673, with BioSample: SAMN41704933, under BioProject PRJNA224116. The assembled genome is available in the NCBI GeneBank under NZ_CP158121 [17].

## Limitations

‘Not applicable’.

## Ethics Statement

Work did not include animal experiments or data collected from social media platforms or human subjects

## CRediT authorship contribution statement

**Amankeldi Sadanov:** Funding acquisition, Conceptualization, Writing – review & editing. **Elvira Ismailova:** Methodology, Software. **Madina Alexyuk:** Investigation, Validation, Writing – original draft, Writing – review & editing. **Olga Shemshura:** Methodology, Software. **Gul Baimakhanova:** Methodology, Software. **Baiken Baimakhanova:** Resources, Formal analysis. **Zere Turlybaeva:** Investigation. **Assel Molzhigitova:** Investigation. **Akmeiir Yelubayeva:** Resources. **Diana Tleubekova:** Resources. **Andrey Bogoyavlenskiy:** Software, Formal analysis, Writing – original draft, Writing – review & editing.

## Data Availability

NCBIComplete genome sequence (Original data).
